# Presymptomatic Diagnosis with MRI and Adequate Treatment Ameliorate the Outcome after Natalizumab-Associated Progressive Multifocal Leukoencephalopathy

**DOI:** 10.3389/fneur.2013.00011

**Published:** 2013-02-18

**Authors:** Hans Lindå, Anders von Heijne

**Affiliations:** ^1^Neurology Unit, Division of Internal Medicine, Danderyd Hospital, Karolinska InstitutetStockholm, Sweden; ^2^Department of Radiology, Danderyd HospitalStockholm, Sweden

**Keywords:** MS, natalizumab, PML, treatment, diagnosis

## Abstract

Natalizumab (Tysabri^®^) is a monoclonal antibody that prevents inflammatory cells from binding to brain endothelial cells and passing into the brain parenchyma. Natalizumab is a highly effective treatment for relapsing-remitting multiple sclerosis (MS). Progressive multifocal leukoencephalopathy (PML) is an opportunistic brain JC virus infection that has been shown to be associated with natalizumab treatment. We describe PML in a patient with MS after 44 monthly infusions of natalizumab. With the aid of a routine Magnetic resonance imaging (MRI) scan, PML was detected before any unambiguous clinical manifestations had emerged. PML was treated with plasma exchange to accelerate removal of natalizumab. Mirtazapine and mefloquine was promptly added and approximately 1 month after plasma exchange, when an immune-reconstitution-inflammatory-syndrome appeared, steroid treatment was initiated. Steroid treatment was then continued until no virus could be detected in the cerebrospinal fluid. The outcome was favorable. We believe that this case clearly illustrates the importance of an early, presymptomatic, detection of PML, and an adequate treatment. We also propose that surveillance with MRI scans, every 3 months after 24 months of treatment, should be performed in JC virus antibody positive natalizumab-treated MS patients in order to detect PML in an early phase.

## Case Presentation

**Figure 1:**
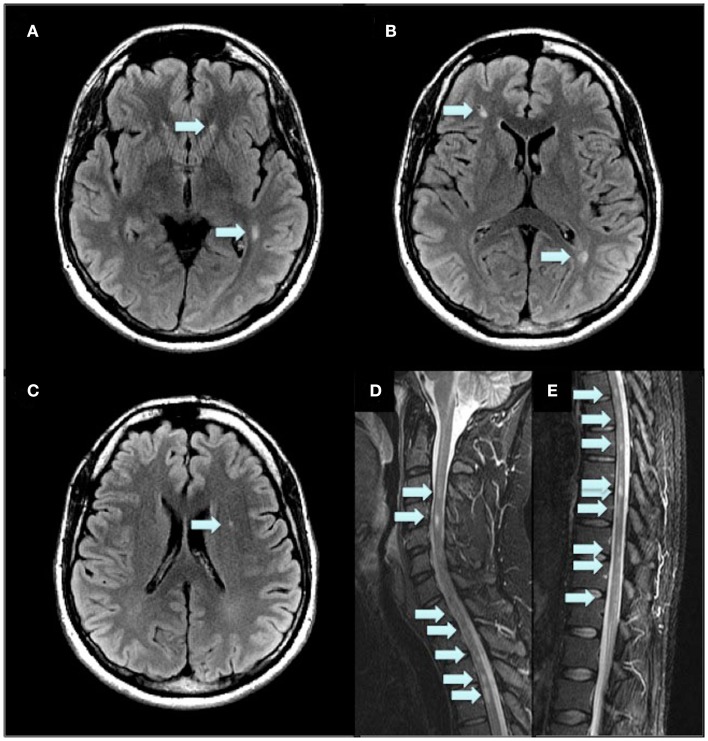
**Initial MRI 2D Flair of the brain (A–C) and FSTIR of the spinal cord (D,E) at the time of MS diagnosis**. Arrows indicate MS lesions.

In February 2008, a 39-year-old man was diagnosed with ulcerative colitis and treatment was initiated with azathioprine. He also suffered from Graves’ disease (causing strabismus and diplopia) and underwent a thyroidectomy. Azathioprine was discontinued in August 2008 due to abnormal liver function. At that time, he began to experience numbness and weakness of both legs and his left arm. Magnetic resonance imaging [MRI; Signa Twinspeed 1.5 T (General Electric)] showed multiple lesions typical for multiple sclerosis (MS) of both the brain (9 lesions) and spinal cord (20 lesions; Figure 1). No gadolinium enhancement was seen. The patient’s tendon reflexes were clearly exaggerated in both legs and he experienced a decreased sensation of vibration. Babinski’s sign was present. He also experienced urinary retention and the need for intermittent catheterization. Analysis of the cerebrospinal fluid (CSF) showed oligoclonal IgG bands. The patient’s score on Kurtzke Expanded Disability Status Scale (EDSS) was 5.5 (on a scale ranging from 0 to 10, with higher scores indicating a greater degree of disability). Thus, within a year, he had been diagnosed with three different autoimmune diseases.

### Natalizumab therapy

**Figure 2:**
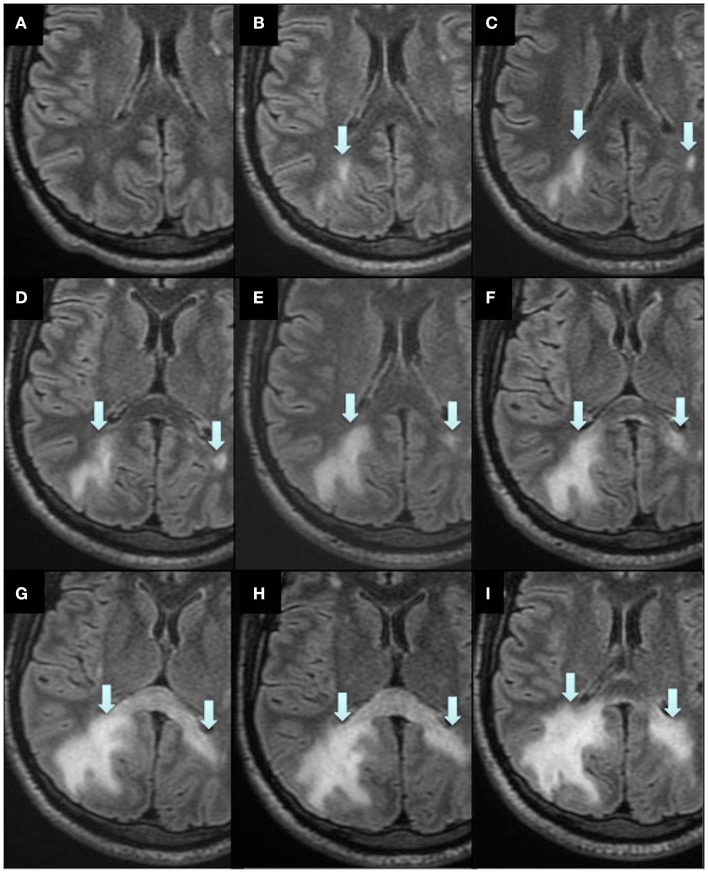
**Magnetic resonance imaging scans during the course of PML and IRIS**. Axial reconstructions from 3D Flair acquisition. Routine MRI 1 year before PML diagnosis **(A)**, when PML was diagnosed **(B)** and subsequent scans directly after plasma exchange **(C)**, 1 week **(D)**, 2 weeks **(E)**, 4 weeks **(F)**, 5 weeks **(G)**, 1.5 months **(H)**, and 2.5 months **(I)** after plasma exchange. Arrows indicate the PML lesions.

**Figure 3:**
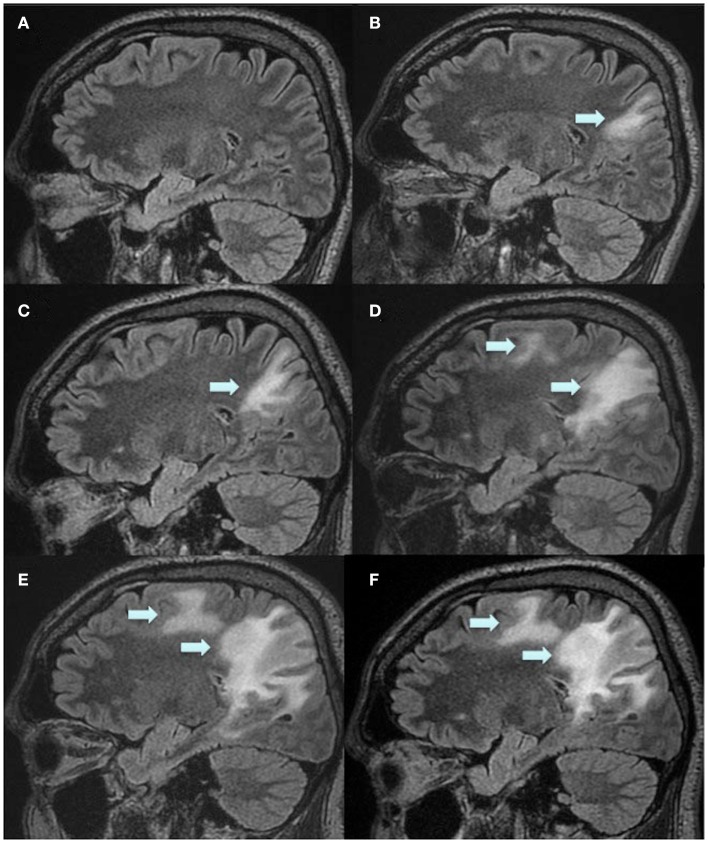
**Magnetic resonance imaging scans during the course of PML and IRIS**. Sagittal reconstructions through the right hemisphere from 3D Flair acquisition. Routine MRI 1 year before PML was diagnosed **(A)**, when PML was diagnosed **(B)** and subsequent scans 2 weeks **(C)**, 1.5 months **(D)**, 2.5 months **(E)**, and 3.5 months **(F)** after plasma exchange. Arrows indicate the PML lesions.

Due to a presumed highly active MS disease, intravenous natalizumab therapy (at a dose of 300 mg every 4 weeks) was initiated already in December 2008. This treatment was also considered effective for his colitis. Despite natalizumab treatment, he developed severe spasticity and pain in his legs, therefore baclofen and duloxetine treatment was initiated in May 2009. At that time, he could still walk short distances with the aid of a cane and EDSS was 6.0. MRI in March 2010 showed no new lesions, and there were no clinical relapses. However, in May 2011, MRI showed a new lesion in the cervical spinal cord and he experienced accentuated spasticity in his legs and left arm. EDSS was now 7.5 and he was confined to a powered wheelchair, but his cognitive functions still seemed intact. He was home living but he needed human help to complete activities of daily living (ADL). In August 2011, he tested positive for JC virus antibodies. In order to treat his severe spasticity, he was treated with nabiximols (Sativex) from February to April 2012. In late April 2012, after 44 natalizumab infusions, a routine MRI scan showed a diffusely demarcated parietal subcortical lesion in the right hemisphere (Figures 2 and 3). At that time, the patient’s EDSS was still 7.5, and he had not experienced any new neurological symptoms. However, he scored only 42 on a Symbol Digit Modalities Test (SDMT; see Discussion). In a test 6 months earlier he had scored 51, and in December 2008, when natalizumab treatment was initiated, he had scored 46. PML was suspected and later confirmed when analysis of the CSF with a polymerase-chain-reaction (PCR) assay for JC virus DNA showed 7100 copies/milliliter. The CSF showed no pleocytosis.

### Treatment of PML

**Figure 4:**
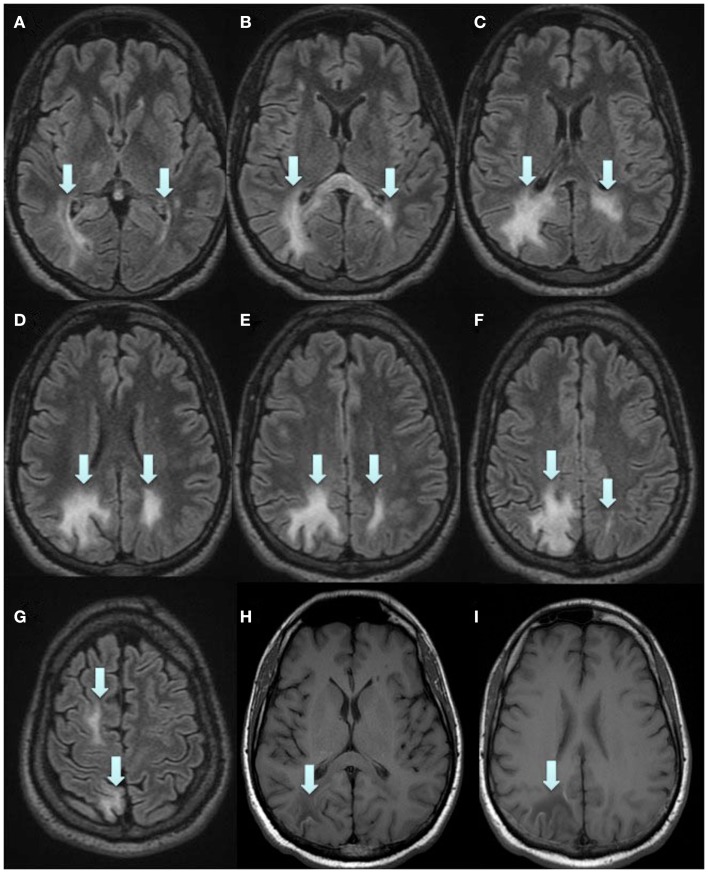
**Final, stable status, 7 months after PML diagnosis**. 3D Flair **(A–G)** and T1w SE **(H,I)**. **(H,I)** Show laminar necrosis in part of the initial lesion. Arrows in **(A–G)** indicate the PML lesions and in **(H,I)** the laminar necrosis.

Plasma exchange was immediately initiated to accelerate removal of natalizumab, in order to restore immune function, with a total of five treatments every second day. Moreover, mefloquine 250 mg was added once daily for three consecutive days followed by once a week, and mirtazapine 30 mg was added once daily. Directly after the plasma exchange, MRI displayed a new PML lesion adjacent to the splenium in the left hemisphere, and the initial lesion in the right hemisphere had increased in size (Figure 2). MRI was performed twice a week to immediately discover any signs of gadolinium enhancement, indicating an immune-reconstitution-inflammatory-syndrome (IRIS) reaction. In mid June, approximately 1 month after the plasma exchange, his legs suddenly became weaker and he complained of headache. IRIS was now suspected and intravenous solumedrole treatment, 1 g daily for five consecutive days, was initiated. However, MRI 2 days later still showed no gadolinium enhancement, perhaps due to the steroid treatment which had already been initiated. At this time, in the left hemisphere, the lesion adjacent to the splenium had further increased in size (Figure 2). In the right hemisphere, the initial lesion had also increased in size, and additional lesions appeared in the superior frontal lobe and splenium (Figures 2 and 3). At no time during the whole course of the disease was any contrast enhancement detected on MRI. Directly after solumedrole, prednisolone at a dose of 30 mg, once daily, was initiated. A new CSF analysis of JC virus DNA in July, 2.5 months after plasma exchange, showed 510 copies/ml and pleocytosis (10 mono). A last CSF analysis in August, 3.5 months after the last plasma exchange, was negative for JC virus DNA, and no pleocytosis was at hand. Prednisolone dose was then tapered off over a period of 4 weeks. He is from October 2012 treated with interferon beta-1a (Rebif^®^ 44 μg). In November 2012, 7 months after PML diagnosis, he scored 35 on SDMT. EDSS was not changed (7.5), and he could still drive his powered wheelchair independently. He is still home living and needs human help for his ADL. However, he complains of increased spasticity in his legs and left arm. He also experienced fatigue. An MRI scan performed in November 2012 displayed laminar necrosis in the initial PML lesion in the right parietal lobe (Figure 4).

## Background

Natalizumab (Tysabri^®^) was introduced for the treatment of highly active relapsing-remitting MS in November 2004. Natalizumab is a monoclonal antibody directed against α4β1 and α4β7 integrins that prevents inflammatory cells from binding to brain endothelial cells and passing into the brain parenchyma (Stüve et al., 2006). Natalizumab is highly effective with a clear reduction in relapse rate, a reduction in new T2-weighted and T1 contrast enhancing lesions (Rudick et al., 2006; Polman et al., 2006). As of December 2012, more than 100,000 patients have been treated with natalizumab worldwide, and among them more than 20,000 with a treatment duration exceeding 4 years (Biogen Idec, data on file). Progressive multifocal leukoencephalopathy (PML) is a rare and often fatal demyelinating disease of the central nervous system (CNS) that occurs in immunocompromised patients as in, for example, myelo- and lymphoproliferative disorders or human immunodeficiency virus (HIV)/AIDS (Koralnik, 2004). The etiological agent is the human JC polyomavirus (JCV) which remains latent in the kidneys and lymphoid organs of infected immunocompetent patients. In infected immunocompromised patients the JCV could mutate and infect oligodendrocytes which causes multifocal demyelination. Another cause of PML is immunosuppressive drug therapy such as natalizumab (Kleinschmidt-DeMasters and Tyler, 2005; Langer-Gould et al., 2005; Yousry et al., 2006; Lindå et al., 2009). As of December 2012, 312 cases of natalizumab-associated PML have been reported (Biogen Idec, data on file). Natalizumab-associated PML infections are lethal in approximately 20% of cases, and among survivors it most often leads to severe disability (Vermersch et al., 2011). Three main risk factors for natalizumab-PML are known, i.e., 1/prior JC virus infection with the presence of anti-JC virus antibodies, 2/prior use of immunosuppressants, and 3/a treatment duration exceeding 24 months (Bloomgren et al., 2012). In the general population with MS, approximately 55% are JC virus antibody positive (Bloomgren et al., 2012). No PML cases have been confirmed in JC virus antibody negative patients. With a positive anti-JC virus antibody status, the incidence of PML is 0.56 (0.36–0.83) per 1000 patients when there are no prior use of immunosuppressants and a treatment duration shorter than 24 months. In contrast, the incidence is 11.1 (8.3–14.5) per 1000 in JC virus antibody positive patients with a prior use of immunosuppressant and a treatment duration exceeding 24 months (Bloomgren et al., 2012).

## Discussion

We describe a patient with natalizumab treatment who, based on findings on a routine MRI scan, was diagnosed with PML before any unambiguous clinical manifestations had emerged. PML is a serious adverse effect with high morbidity and mortality rates complicating natalizumab treatment. Clinical vigilance is always important for the diagnosis, but this case clearly illustrates the importance of an early detection with MRI and an adequate treatment to accomplish a favorable outcome. A few short case reports have recently been published of PML discovered by MRI in the presymptomatic phase (Blair et al., 2012; Phan-Ba et al., 2012a). In these cases, clinical outcome was also favorable, but in our case poor prognosis would be predicted according to factors reported as associated with poorer survival, i.e., 1/older age at PML diagnosis, 2/higher pre-PML EDSS, and 3/widespread PML extension on MRI (Biogen Idec, data on file). This is in line with emerging evidence that early diagnosis of PML, before any symptoms develop, is of importance for the outcome (Phan-Ba et al., 2012b). Thus, we are convinced that MRI scans (FLAIR) every 3 months after 24 months of natalizumab treatment, when risk to develop PML is increased, should be recommended in all JC virus positive MS patients (Table 1). It is probably even more important with frequent MRI scans among natalizumab treated MS patients with a high EDSS score, as shown in this case, while it is more difficult to discover a subtle clinical worsening among these patients. Our patient also had a prior use of immunosuppressant, which further emphasize the importance of frequent MRI scans in such cases. The importance of frequent MRI scans was also obvious in our first case of natalizumab-associated PML, which also was the first case described with natalizumab monotherapy (Lindå et al., 2009). In that case, we could demonstrate that signs of PML on MRI was at hand already 3 months before any symptoms had appeared (Lindå et al., 2009). In the present case, regular MRI scans every 3 months, most probably would have discovered the PML infection even earlier, which possibly would have led to an even more favorable outcome. The present case also demonstrates the importance of frequent SDMT, while the patient’s score was clearly lower at PML diagnosis compared to 6 months earlier. SDMT measures cognitive processing speed and visual working memory. It is a performance measure that requires patients to visually scan a key of number/symbol pairings and then voice the correct number for randomly presented symbols as rapidly as possible (Smith, 1982). The outcome measure is the number of correct responses in 90 s. SDMT is strongly associated with MS lesion burden and cortical and deep gray matter atrophy (Houtchens et al., 2007; Benedict et al., 2006). However, there is often some variation in scores between tests in the same patient over time, which makes it difficult from one test to determine whether it is a temporary decline in score or a first indication of PML. A monthly assessment should then be necessary to screen for cognitive impairments, instead of the usually scheduled every 6 months. The sensitivity and specificity of SDMT with regard to early discovery of PML remains to be evaluated. The efficacy of plasma exchange in the treatment of natalizumab-associated PML has already been shown in several case reports (Lindå et al., 2009; Khatri et al., 2009; Schroder et al., 2010; Wenning et al., 2009). Also in the present case, plasma exchange was initiated immediately after diagnosis of PML to quickly remove natalizumab, and thus restore immune function. Mefloquine was given directly after plasma exchange. Mefloquine has been shown to inhibit JC viral replication in cells *in vitro* (Brickelmaier et al., 2009), and to exert beneficial clinical effects on PML infections (Beppu et al., 2012). Treatment with mirtazapine (at a dose of 30 mg per day) was also initiated after plasma exchange to probably further inhibit viral spread by blocking the 5-HT2 receptor, the proposed viral coreceptor for cellular infection (Aksamit, 2008). Close clinical vigilance and frequent MRI scans was then performed in order to detect the first symptoms suggestive of IRIS. IRIS is a well known condition from patients with HIV infections suffering from PML, where it occurs in parallel with immune recovery after highly active antiretroviral therapy (Venkataramana et al., 2006). It is caused by the presumed immune attack against the JC virus, resulting in an overwhelming inflammatory response. This inflammatory attack is then believed to increase the local damage. Thus, IRIS itself can probably cause significant morbidity and mortality and should be treated with steroids (Tan et al., 2009). However, the use of steroids for IRIS has been challenged (Berger, 2009). IRIS can be detected either by gadolinium enhancement on MRI or by a worsening of symptoms. In both this case and in our first case of natalizumab-associated PML (Lindå et al., 2009), IRIS occurred within 1 month from plasma exchange and was accompanied by headache, possibly a consequence of meningeal irritation. In order not to interfere with the initial immune recovery, we waited with steroids until a presumed IRIS was at hand. The treatment with high dose steroids intravenously was followed by an oral steroid course for several months until PCR for JC virus DNA in CSF showed negative results.

**Table 1:** **Proposed scanning surveillance with a complete MRI protocol yearly and MRI scans with only 3D FLAIR images with 3 months interval**.

MRI	Yearly	Month 3	Month 6	Month 9
Protocol	SAG 3D FLAIR	SAG 3D FLAIR	SAG 3D FLAIR	SAG 3D FLAIR
	Ax T2 FSE			
	Ax DWI			
	Ax T1 SE			
	Ax T1 SE w CM			

*FLAIR, fluid attenuated inversion recovery; FSE, fast spin echo; DWI, diffusion weighted imaging; CM, contrast media (gadolinium)*.

## Concluding Remarks

Our patient’s history clearly illustrates the importance of frequent MRI scans to detect PML in an early phase, thus anticipating a more favorable outcome. We propose that MRI scans should be performed every 3 months after 24 months of natalizumab treatment among JC virus antibody positive MS patients. We also propose that natalizumab-associated PML should be treated according to what is described in this case, i.e., plasma exchange followed by mirtazapine and mefloquine. Steroid treatment should be initiated when IRIS emerges and continued until no JC virus DNA can be detected in the CSF. Thus, we herein provide a possible algorithm for the diagnosis and treatment of natalizumab-associated PML.

## Conflict of Interest Statement

The authors declare that the research was conducted in the absence of any commercial or financial relationships that could be construed as a potential conflict of interest.
